# Patient safety culture and associated factors among health care providers in government and private hospitals, Bahir Dar City Northwest, Ethiopia, 2022: a comparative cross-sectional study

**DOI:** 10.1186/s12913-023-09770-4

**Published:** 2023-07-18

**Authors:** Tezeta Ayanaw, Eshetu Abera Worede, Mekuriaw Alemayehu, Walelegn Worku, Giziew Abere, Bikes Destaw Betew

**Affiliations:** 1 Gondar Town Labour and Social Affairs office, Gondar, Ethiopia; 2 Department of Environmental and Occupational Health and Safety, Institute of Public Health, University of Gondar, Gondar, Ethiopia

**Keywords:** Safety culture, Patient safety, Private and public healthcare providers, The risk factor

## Abstract

**Background:**

Patient safety in a healthcare setting is now a major global concern. Millions of people suffer disabling injuries or death directly related to medical care errors, particularly in developing countries. Evidence about patient safety culture in Ethiopia is limited. Therefore, this study was designed to assess the level of patient safety culture and associated factors among healthcare providers in government and private healthcare providers.

**Methods and materials:**

Institution based cross-sectional study was conducted from May to June 30, 2022. Self-administered hospital survey on Patient Safety Culture (HSOPSC) tool was used to select 448 study participants. Epi Data version 4.6 and SPSS version 26 were used for data entry and analysis. Chi-square test, Bi-variable, and multivariable logistic regressions were done to determine the association between the independent and outcome variable.

**Result:**

A total of 448 healthcare providers with a response rate of 99.6% participated. The prevalence of good patient safety culture was 50.9%( 95%CI: 46.2, 55.6%). Patient safety culture difference was observed between government and private healthcare providers (× 2 = 22.6, df = 1, *p* = 0.000). Type of hospitals (AOR = 0.37(95% CI:(0.21, 0.68), profession (AOR = 2.16 (95% CI:(1.02,4.62), job satisfaction (AOR = 0.19,95%CI:(0.12,0.30), participated in patient safety programs(AOR = 2.69:(95%CI:1.53,4.75), providing necessary equipment and materials (AOR = 2.05(95%CI: 1.18,3.55%), and work shift (AOR = 0.47( 95%CI: 0.25,0.93) were found significantly associated with good patient safety culture among healthcare providers.

**Conclusion:**

The prevalence of good patient safety culture was relatively low. Patient safety culture difference is observed between government and private healthcare providers. Type of hospitals (public or private), profession, job satisfaction, participation in patient safety programs, providing necessary equipment and materials, and work shifts were associated factors for patient safety culture. Therefore, it is better to design patient safety improvement strategies for both government and private healthcare providers.

## Introduction

Patient safety culture refers to the values, beliefs, and norms that are shared by healthcare practitioners and other staff throughout the organization that influences their actions and behaviors related to patient safety in the organization that support and promote patient safety [1]. Institute of Medicine (IOM) and the World Health Organization (WHO) defined patient safety as the prevention of harm to patients [2]. The absence of preventable harm to a patient during the process of healthcare, and the reduction of risk of unnecessary harm associated with healthcare to an acceptable minimum [3]. Global efforts to reduce the burden of patient harm have not been achieved over the past 15 years despite pioneering work in some healthcare settings [4].

A recent global study found that there is an urgent need to promote a patient safety culture [5]. Various literature from high-income countries shows that a significant number of patients are harmed during healthcare processes, resulting in either increased medical costs, extended hospital stays, permanent disabilities, or even death [6]. Recent studies also revealed that medical errors are the third leading cause of death in the United States after cancer and heart disease [7]. Globally, from 421 million hospitalizations, 42.7 million adverse events occur in patients [6, 8], and low and middle-income countries account for two-thirds of all those adverse events [9]. World Alliance and the WHO report showed that to give attention to sub-Saharan African countries for urgent understanding, action, and improvement of patient safety culture [9, 10].

A study in Iran about patient safety culture found a more positive response in public hospitals (65.5%) than a private hospitals (58.3%) [11]. Several factors affect patient safety culture including sex, age [12], religion, educational level, marital status, monthly income, and work experience of healthcare providers [13]. Work-related and facility-based variables such as type of hospital and work shift [14], types of profession, and participation in patient safety programs [15]. A study conducted in Ghana found two out of twelve patient safety culture dimensions recorded high positive response rates (≥ 70%), and three patient safety culture dimensions such as staffing, non-punitive response to error, and frequency of events reported recorded low positive response rates [16].

Providing feedback on errors and requirements for frequent incident reporting, and patient information exchange was necessary to promote the patient safety culture [14]. Developing a positive patient safety culture is a crucial element in the improvement of patient safety in a healthcare organization [17, 18]. Achieving a culture of patient safety requires an understanding of the values, beliefs, and norms about what is important in an organization, and what attitudes and behaviors related to patient safety are supported, rewarded, and expected [19]. In Ethiopia, there is a national healthcare quality strategy that focused on patient safety and this research will contribute to this strategy for patient safety culture improvement [20]. Therefore, this study aimed to assess the level of patient safety culture and associated factors among healthcare providers in government and private healthcare providers.

## Methods and materials

### Study setting, design, and period

An institutional-based cross-sectional study was conducted from May 25 to June 31/2022 in government and private hospitals, in Bahir Dar city. The City has one specialized, one referral, one primary government hospital, and four private healthcare hospitals. All healthcare providers who were working at government and private hospitals were the source population.

### Inclusion and exclusion Criteria

All healthcare providers who have worked at least 6 months before the data collection period were included in the study whereas healthcare providers that were on long-term training and extended leave and healthcare providers working part-time weekly and participants not willing to participate were excluded [21].

### Sample size determination and sampling procedures

The sample size was done for both the first and second objectives. For the first objective (prevalence) sample size was done by considering the following assumptions. The margin of error (d) is 5%. The study considered a 44.8% prevalence of patient safety culture from a previous study at public hospitals in Dessie Town, 2019 [13].

$$\mathrm n=\left(\mathrm{Za}/2\right)^2\mathrm x\;\mathrm p\left(1-\mathrm p\right)/\mathrm d^2$$

Where: n = minimum sample size, Zα/2 = (1.96)^2^.

n = (1.96)^2^ × 0.448 (1– 0.448))/0.05^2^ = 380, so by adding 10% non-response rate (380 + 38) = 418 for the first objective. For the second objective (associated factors) sample size was done using open Epi Info version 7 with considering the following assumptions, power 80%, 95%CI, and risk factors such as age, types of hospitals, and ward type from previous studies. The sample size for the second objective was 450. The sample size for the second objective was higher than the sample size for the first objective. Therefore, the final sample size for this study was the maximum sample size (*n* = 450) which is calculated for the second object.

### Sampling procedures

**Fig. 1:**
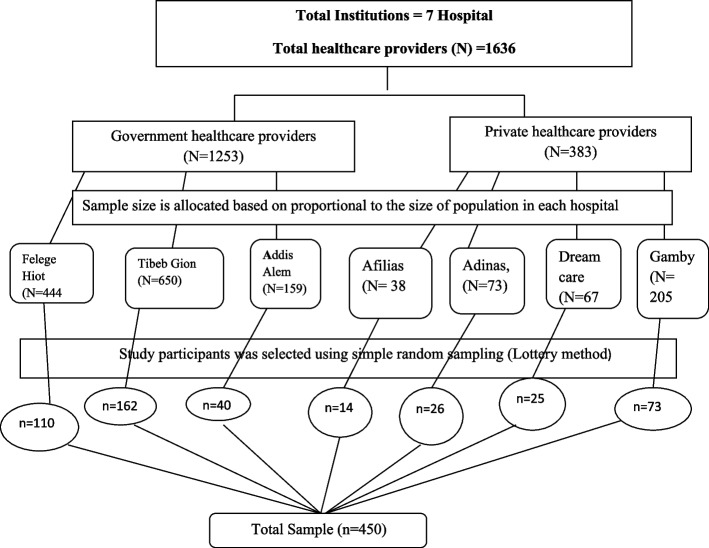
Schematic presentation of sampling procedure among healthcare providers in governmental and private hospitals, Bahir Dar City Northwest, Ethiopia, 2022

The sample size was allocated based on proportional allocation to the total healthcare professionals found in each hospital (government and private). Then, the study participant was selected using simple random sampling (lottery method) from each hospital. The list of study participants was get from the human resource office of each hospital (Fig. 1).

#### Operational definitions

##### Patient safety

Is defined as the absence of preventable harm to a patient during the process of healthcare and the reduction of risk of unnecessary harm associated with healthcare to an acceptable minimum [3].

##### Good and poor patient safety culture

Positive responses in positively worded survey items were agree/strongly agree and positive responses in negatively worded items were disagree/strongly disagree after computing the final score it was dichotomous and the study participants scored ≥ 75% hospital Survey on Patient Safety Culture (HSOPSC)were considered having good patient safety culture, and whereas a score of < 75% hospital Survey on Patient Safety Culture (HSOPSC) questions were having poor patient safety culture [13].

##### Job satisfaction

Ten Likert scale items were used. When a healthcare provider responds and scores above the mean score they are categorized as satisfied, whereas respondents who scored below the mean score were classified as unsatisfied [22].

### Data collection tools and procedure

Background characteristics of study participants, and working condition factors such as type of hospital (teaching/referral, district), work experience, working hours per day, per week, staff position, patient safety training, participation in the patient safety program, and adverse event reporting, communication about errors, Frequency of events reported, Management support. To assess the level of patient safety culture the Hospital Survey on Patient Safety Culture (HSOPSC) tool was adopted from Healthcare Research and Quality (AHRQ) [23], and its validated [24]. The tool was designed to assess hospital staff opinions about patient safety issues, medical errors, and event reporting and it has 44 items that measure 14 dimensions or composites of patient safety culture. The response to each item in the questionnaire was assessed using a 5-point Likert scale of agreements (from 1: “Strongly disagree” to 5: “Strongly agree”) or frequency (from 1: “Never” to 5: “Always”), and the tool was included both positively and negatively worded items.

Job satisfaction of health professionals was assessed using ten items with a Likert scale consists five options (1 – very dissatisfied, 2 – dissatisfied, 3 – neutral, 4 – satisfied, and 5 – very satisfied). The response scales were added and summarized out of 50. Participants who scored above the mean score are categorized as satisfied, whereas respondents who scored below the mean score were classified as unsatisfied [25].

### Data quality control

Two-day training was given for data collectors and supervisors. A pre-test was done on five percent of the study population. To ensure the completeness, accuracy, and consistency of the collected data supervision was done each data collection day. The validity and reliability were assessed using the Cronbach alpha value (*p* = 0.87).

### Data management and statistical analysis

The collected data was entered into Epi-Data version 4.6 and exported to SPSS version 26 software for further analysis. Descriptive statistics were computed. Chi-square, ANOVA, binary, and multivariable logistic regression analyses were performed. Variables that showed association with a dependent variable in the bivariable analyses at *p* < 0.2 were exported to the multivariable logistic regression model. *P*-value ≤ 0.05 and 95% CI were used to define statistical significance. Hosmer and Lemeshow's goodness-of-fit test was checked (*p*-value = 0.268).

## Results

### Socio-demographic characteristics of study participants

A total of 448 healthcare providers with a response rate of 99.6% participated. The mean age of the respondents was 34 ± (6) years. More than half (54.7%) of the study participants were males. Nearly three fourth (67.2%) of study participants had a Bachelor's degree (Table 1).

**Table 1:** Socio-demographic characteristics of health care providers in Bahir Dar City North West, Ethiopia, 2022 (*n* = 448)

Variable	Category	Response	
		Frequency(n)	Percent (%)
Age	< 34	254	56.7
	34–38	91	20.3
	> 38	103	23.0
Sex	Male	245	54.7
	Female	203	45.3
Marital status	single	166	37.1
	Married	270	60.3
	windowed	12	12.7
Educational status	Diploma	78	17.4
	Bachelor degree	301	67.2
	Masters	53	11.8
	Doctorate( PhD)	16	3.6
Work experience	< 3	175	39.1
	3–4	54	12.1
	5–8	111	24.8
	> 8	108	24.1

### Facility and work-related characteristics of study participants

Nearly three-quarters (69.2%) of study participants were from government healthcare institutions. In this study, more than half (52.7%) of healthcare providers were satisfied with their jobs. The majority (65.4%) of healthcare providers participated in patient safety programs. Furthermore, the majority (82.1%) of healthcare providers had worked fewer than 48 h per week (Table 2).

**Table 2:** Facility and work-related characteristics of health care providers, 2022 (*n* = 448)

Variables	Frequency(n)	Percent (%)
Workers from Hospital Type		
Government	310	69.2
Private	138	30.8
Perceived job satisfaction		
Satisfied	236	52.7
Not satisfied	212	47.3
Participated in a patient safety program		
Yes	293	65.4
No	155	34.6
Reporting Adverse events		
yes	237	52.9
No	211	47.1
Participated in a patient safety training program		
yes	154	34.4
No	294	65.0
Hours of work per week		
< 48 h	368	82.1
≥ 48 h	80	17.9
Do you have shift work		
yes	228	50.9
No	220	49.1
Work shift types		
Every 8 h	110	24.6
Regular(day)	264	58.9
Night shift(5 pm-8am)	74	16.5

### Patient safety culture dimension-related characteristics of healthcare provider

According to this study, nearly half (46.4%) of healthcare providers have positive patient safety perceptions. Teamwork across hospital departments was rated positively by half of the respondents (50%). The patient safety grade within the unit/department received a higher score than the rest of the patient safety culture dimension variables with the frequency of event reporting receiving the least positive response (41.5%).

### Patient safety culture in private and government healthcare providers

The level of good patient safety culture was 50.9%( 46.2, 55.6). The patient safety culture is differed significantly between government and private healthcare providers. (X2 = 22.6, df = 1, *p* = 0.000) (Table 3).

**Table 3:** Patient safety culture between government and private healthcare providers (*n* = 448), 2022

Types of the healthcare provider	Patient safety culture		*p*-value
	good	Poor	
Government	181(40.4%)	129(28.8%)	< 0.05
Private	47(10.5%)	91(20.3%)	
Total	228(50.9%)	220(49.1%)	

### The mean scores of patient safety culture dimension measures based on the types of hospital

There was a significant difference in the mean scores of the patient safety culture dimension variables between private and government hospitals (*p* < 0.05). However, there was no statistically significant difference in the mean scores of sub-patient safety culture dimension measures such as no punitive response to errors, and hospital handoffs and transition (*p* > 0 0.05) (Table 4).

**Table 4:** Patient Safety Culture dimension Mean Scores based on the type of Hospital, northwest Ethiopia (*n* = 448)

Patient safety culture dimensions variable	Types of hôpital		*p*-value
	Government (x ± SD)	Privat (x ± SD)	
The overall perception of patient safety culture	1.59 ± 0.49	1.41 ± 0.49	0.001
The frequency of the event reported	1.68 ± 0.46	1.36 ± 0.48	0.001
Supervisor/manager expectations and action promoting patient safety	1.57 ± 0.49	1.26 ± 0.44	0.001
Organizational learning to continuously improve	9.98 ± 2.8	10.87 ± 3.3	0.001
Teamwork within department	1.47 ± 0.49	1.36 ± 0.48	0.032
Communication openness	1.50 ± 0.50	1.33 ± 0.47	0.001
Feedback and communication about error	1.45 ± 0.49	1.34 ± 0.47	0.028
No punitive response to errors	1.54 ± 0.49	1.44 ± 0.49	0.059*
Staffing	1.48 ± 0.49	1.44 ± 0.49	0.001
Hospital management support for patient safety	1.57 ± 0.40	1.32 ± 0.47	0.001
Teamwork across the hospital department	1.55 ± 0.49	1.36 ± 0.48	0.001
Hospital handoffs and transition	1.46 ± 0.49	1.41 ± 0.49	0.28*
The overall patient safety culture	1.42 ± 0.5	1.65 ± 0.47	0.001

*p*- Value obtained from ANOVA test

^*^No statistical significance

### Factors associated with patient safety culture among healthcare providers in Bahir Dar City, northwest, Ethiopia 

In bivariable logistic regression analysis, fourteen variables such as age, educational status, work experience, hospital type(tier)( Referral and primary) and hospital type(private and government), profession, job satisfaction, participation in the patient safety program, taking patient safety training, reporting adverse events, shift work, providing necessary equipment and materials at the time of giving care, hospital management blame to medical errors, and hospital management encourage reporting events, were candidate (*p* ≤ 0.2) for multivariable logistic regression. After controlling confounder variables in multivariable logistic regression analysis, only variables such as profession, hospital type (government and private), job satisfaction, participation in the patient safety program, providing necessary equipment and materials at the time of giving care, and work shift were found significantly associated with patient safety culture.

The odds of good patient safety culture were 2.16 times higher among Midwives when compared to Physician healthcare professionals (AOR = 2.16(95%CI:(1.02, 4.62). In this study, good patient safety cultures were 63% times lower among private healthcare providers when compared to government healthcare providers (AOR = 0.37(95%CI:(0.21,0.68). The level of good patient safety culture was 81% times lower among healthcare providers who were not satisfied with their jobs when compared to their counterparts (healthcare providers satisfied with their job)(AOR = 0.19,95%CI: (0.12,0.30).

According to this study, good patient safety culture was 2.69 times higher among healthcare providers who participated in patient safety programs when compared to their counterparts (AOR = 2.69(95%CI: 1.53, 4.75). The odds of good patient safety culture were 2.05 times higher when providing necessary equipment and materials at the time of giving care (AOR = 2.05(95%CI: 1.18, 3.55). Furthermore, good patient safety cultures were 53% times lower among healthcare providers that work regular(day) work shifts when compared to healthcare professionals that work in the night shift work (AOR = 0.47(95%CI: (0.25, 0.93) (Table 5).

**Table 5:** Multivariable logistic regression analysis and factors associated with patient safety culture among healthcare providers in Bahir Dar City Northwest Ethiopia, 2022, (*n* = 448)

Variable	Patient safety culture		COR(95%CI)	AOR (95%CI)	*P*-value
	Good	poor			
Age					
< 34	135	119	1	1	
34–38	46	45	0.91(0.56,1.45)	1.046(0.55,1.96)	0.88
> 38	47	56	0.74(0.46,1.17)	1.10(0.57,2.11)	0.77
Educational status					
Diploma	33	45	1	1	
Bachelor degree	158	143	1.51(0.91,2.49)	0.92(0.48,1.73)	0.79
Masters	32	21	2.07(1.02,4.22)	1.42(0.58,3.47)	0.43
Doctorate( PhD)	5	11	0.62(0.19,1.95)	1.05(0.27,4.06)	0.94
Work experience					
< 3	102	73	1	1	
3–4	20	34	0.42(0.22,0.78)	1.15(0.56,2.33)	0.69
5–8	51	60	0.60(0.37,0.98)	0.43(0.18,1.03)	0.60
> 8	55	53	0.74(0.45,1.20)	0.71(0.53,1.43)	0.33
Profession					
physician	39	44	1	1	
Midwives	62	36	1.94(1.07,3.52)	**2.16(1.02,4.62)**	0.046
laboratory	37	33	1.26(0.66,2.39)	2.01(0.88,4.61)	0.09
Others(nurs, pharm)	90	107	0.94(0.56,1.58)	1.49(0.77,2.86)	0.23
Workers working hospital type					
Referral	172	142	1	1	
Primary	56	77	0.60(0.39,0.90)	1.38(0.76,2.49)	0.28
Hospital types					
Government	181	129	1	1	
Private	47	91	0.36(0.24,0.55)	**0.37(0.208,0.68)**	0.001
Job satisfaction					
Satisfied	169	67	1	**1**	
Not satisfied	59	153	0.15(0.10,0.23)	**0.19(0.12,0.30)**	0.001
Participated in a patient safety program					
No	121	172	1	**1**	
Yes	107	48	3.16(2.09,4.78)	**2.69(1.53,4.75)**	0.001
Reporting adverse Events					
yes	96	141	1	1	0.059
No	132	79	2.45(1.67,3.59)	1.65(0.98,2.75)	0.059
Taken any patient safety training					
Yes	68	86	1	1	
No	160	134	1.51(1.02,2.24)	0.83(0.48,1.42)	0.48
Providing necessary equipment and material at the time of giving care					
No	76	139	**1**	**1**	
yes	152	81	3.43(2.33,5.06)	**2.05(1.18,3.55)**	0.01
shift work					
Every 8 h	59	51	0.83(0.46,1.51)	0.75(0.35,1.59)	0.45
Regular( day)	126	138	0.65(0.39,1.10)	**0.47(0.25,0.93)**	0.03
Night(5 pm-8am(	43	31	1	1	0.44
Hospital management blames medical errors					
Yes	122	143	1	0.95(0.57,1.59)	0.86
No	106	77	1.61(1.10,2.36)	0.87(0.53,1.45)	0.6
Hospital management encourages reporting events					
yes	106	141	1	1	0.59
No	122	79	2.05(1.41,3.00)	0.87(0.53,1.45)	0.86

Hosmer and Lemshaw goodness of fit; 0.268, 1 = Reference

## Discussion

Patient safety in the healthcare setting is now a major global concern, and millions of people suffer disabling injuries and deaths related to medical care errors. This study was designed to assess the level of patient safety culture and associated factors among government and private healthcare providers in Bahir Dar City, Amhara region, Ethiopia.

Based on this study, the overall prevalence of good patient safety culture was 50.9%( 46.2, 55.6%). This finding was consistent with the studies conducted in Yemen (46%), Egypt (46.56%), and Jimma (46.7%) [26–28]. The possible justification for this similarity could be similar socio-demographic characteristics of respondents and cross-sectional study design and similar study tools used for outcome variables such as HSOPSC tools in Jimma, Yemen, and this study. On the other hand, this finding is higher than the studies conducted in Dessie (44.8), Bale zone (44%), and Gondar (45.3%) [13, 29, 30]. But, the finding of this study is lower than a study conducted in south India among healthcare Providers in tertiary care hospitals 58% [31]. The possible reason for this discrepancy could be the difference in study design used, sample size difference, the method followed, and the study period and area.

Regarding factors associated with good patient safety culture, the odds of good safety culture were higher among midwives professionals when compared to a physician. The possible justification might be that midwives may give attention to their attendants due to government support to give attention to maternity to prevent unwanted outcomes and different supportive training might influence good patient handling practices of midwives when compared to physicians. In this study, the odds of good patient safety culture were lower among private healthcare providers when compared to government healthcare providers. This finding is consistent with the study conducted in Peru and the average percentage of positive responses to the patient safety culture was higher among government healthcare providers (65.5%) than private healthcare providers (58.3%) [32]. But this finding is contradictory to the study finding in Iran and the average of positive scores in patient safety culture was higher for private (83%), than for public hospitals (78%) [33]. This discrepancy might be due to in Iran there might be strong auditing and monitoring mechanism in private healthcare providers than the government healthcare providers, but in our country, in government hospitals, there might be had strong auditing system for patient safety culture, especially in infection prevention and control (IPC) strategy.

In this study, the other significant variable with patient safety culture was job satisfaction, and the odds of good patient safety culture were lower among healthcare providers who were not satisfied with their jobs when compared to their counterparts (satisfied healthcare providers). The possible reason might be that those health care providers, who were satisfied with their job, love their profession and work enthusitively, welcome patients friendly, and have good teamwork within and across organizations. Another important significant variable associated with good patient safety culture was participation in the patient safety program, and good patient safety cultures were higher among healthcare providers who participated in patient safety programs. This finding is consistent with studies conducted in Southeast Ethiopia, Bale zone, [34], southwest, Ethiopia Gondar [35], and in the UK, that participants who did not attend patient safety courses had significantly lower perceptions of patient safety than those who did attend patient safety courses [36].

Moreover, good patient safety culture was higher among healthcare providers that provide necessary equipment and materials at the time of giving care as compared to their counterparts. This finding is consistent with the study conducted in Gondar [30]. The possible reason might be that lack of necessary materials and equipment might become a barrier to delivering health care services which might affect patient safety culture. Furthermore, good patient safety culture was significantly associated with shift work, and the odds of good patient safety culture were lower among healthcare providers working regular (day) work shifts when compared to healthcare providers working in the night shift program. This finding is consistent with a study conducted in Iran which stated that work-shift hours influenced patient safety culture [14]. The possible justification might be in a day shift there may be a workload due to high patient follow in the daytime than at night time and this might cause the unsafe act to do unwanted medical errors but on the night shift, there might be low patient follow and the healthcare provider may work his job with conscious and can reduce medically related errors.

### Limitations of the study

The data were collected through a self-administered questionnaire, a real observation of practice was not done and there could be a possibility of introducing social-desirability bias.

## Conclusion

The overall prevalence of good patient safety culture was low. The patient safety culture dimension was significantly different between government and private health providers except for no punitive response to error, and hospital handoffs and transition of sub-dimension of patient safety culture. Type of hospitals (private and government), level of job satisfaction, types of profession, participation in patient safety programs, and providing necessary equipment and materials at the time of giving care and work shift were found significant associations with patient safety culture.

## Data Availability

The datasets used for the current study are available from the corresponding author upon reasonable request.

## Abbreviations

AOR: Adjusted odds ratio
COR: Crude odds ratio
CI: Confidence interval
ETB: Ethiopian Birr
HSOPSC: Hospital Survey on Patient Safety Culture
IPC: Infection prevention and control
PPE: Personal protective equipment
SD: Standard deviation
SPSS: Statistical package for social science
